# *CDKN2A* gene inactivation in epithelial sporadic ovarian cancer

**DOI:** 10.1038/sj.bjc.6690621

**Published:** 1999-08

**Authors:** D Niederacher, H-Y Yan, H-X An, H G Bender, M W Beckmann

**Affiliations:** Department of Obstetrics and Gynaecology, Heinrich-Heine-University, Moorenstr. 5, Düsseldorf, 40225, Germany; Department of Obstetrics and Gynaecology, The Third Affiliated Hospital of Hebei Medical University, 16 Weiming Street, Shijiazhuang, 050051, People's Republic of China

**Keywords:** *CDKN2A* gene, LOH, 9p21–22, RT-PCR, immunohistochemistry, ovarian cancer

## Abstract

The tumour suppressor gene *CDKN2A*, located on chromosome 9p21, encodes the cell cycle regulatory protein p16. Inactivation of the *CDKN2A* gene could lead to uncontrolled cell growth. In order to determine the role of *CDKN2A* in the development of sporadic ovarian cancer, loss of heterozygosity at 9p21–22, homozygous deletion, mutation and methylation status of the *CDKN2A* gene as well as *CDKN2A* expression were examined in a panel of serous papillary ovarian cancer. The frequency of loss of heterozygosity (LOH) for one or more informative markers at 9p21–22 was 65% (15/23). The most common deleted region was located between interferon (IFN)-α and D9S171. Homozygous deletions and mutations of the *CDKN2A* gene were not found. There was no evidence of methylation in exon 1, but methylation in exon 2 of *CDKN2A* gene was found in 26% (6/23). Absence of *CDKN2A* gene expression was shown in 27% (6/22) at mRNA level and 21% (4/19) at protein level. These data suggest that the *CDKN2A* gene is involved in the tumorigenesis of ovarian cancer, but the mechanisms of *CDKN2A* gene inactivation in serous papillary ovarian cancer remains unclear. © 1999 Cancer Research Campaign


*British Journal of Cancer* (1999) **80**(12), 1920–1926
© 1999 Cancer Research Campaign
Article no. bjoc.1999.0621

### *CDKN2A* gene inactivation in epithelial sporadic ovarian cancer

**D Niederacher[1], H-Y Yan[1,2], H-X An[1], HG Bender[1] and MW Beckmann[1]**

[1]Department of Obstetrics and Gynaecology, Heinrich-Heine-University, Moorenstr. 5, 40225 Düsseldorf, Germany; [2]Department of Obstetrics and Gynaecology, The Third Affiliated Hospital of Hebei Medical University, 16 Weiming Street, 050051 Shijiazhuang, People's Republic of China

**Summary** The tumour suppressor gene *CDKN2A*, located on chromosome 9p21, encodes the cell cycle regulatory protein p16. Inactivation of the *CDKN2A* gene could lead to uncontrolled cell growth. In order to determine the role of *CDKN2A* in the development of sporadic ovarian cancer, loss of heterozygosity at 9p21–22, homozygous deletion, mutation and methylation status of the *CDKN2A* gene as well as *CDKN2A* expression were examined in a panel of serous papillary ovarian cancer. The frequency of loss of heterozygosity (LOH) for one or more informative markers at 9p21–22 was 65% (15/23). The most common deleted region was located between interferon (IFN)-α and D9S171. Homozygous deletions and mutations of the *CDKN2A* gene were not found. There was no evidence of methylation in exon 1, but methylation in exon 2 of *CDKN2A* gene was found in 26% (6/23). Absence of *CDKN2A* gene expression was shown in 27% (6/22) at mRNA level and 21% (4/19) at protein level. These data suggest that the *CDKN2A* gene is involved in the tumorigenesis of ovarian cancer, but the mechanisms of *CDKN2A* gene inactivation in serous papillary ovarian cancer remains unclear.

**Keywords:** *CDKN2A* gene; LOH; 9p21–22; RT-PCR; immunohistochemistry; ovarian cancer

Ovarian cancer (OC) is the leading cause of death in women with gynaecological malignancies. In the USA in 1996 approximately 24 000 OC were diagnosed with 13 600 deaths, and in Germany 6400 cases with a similar death rate (Beckmann et al, 1996*a*). Because of the absence of early symptoms the majority of patients are diagnosed with advanced stages. Existing therapeutic measures are often ineffective and the prognosis of the patients is poor.

Molecular genetic alterations play a key role in carcinogenesis. Understanding genetic events that lead to initiation and progression of ovarian cancer remains an important challenge in gynaecological research.

Multiple genetic changes, including activation of proto-oncogenes and inactivation of tumour suppressor genes, are involved in the genesis and progression of human cancers. Some associations of genetic alterations with OC development has been reported. Among them the involvement of chromosome 9 is very noticeable. It was first reported by Cliby et al (1993) and confirmed by others using loss of heterozygosity (LOH) analysis (Chenevix-Trench et al, 1994; Osborne and Leech, 1994). The most frequent LOH region on chromosome 9p is next to D9S171 (Chenevix-Trench et al, 1994; Shih et al, 1997). This indicates a potential tumour suppressor gene in this region involved in the tumorigenesis of ovarian cancer.

The *CDKN2A* (also called *CDKN2*, *MTS1*, *CDK4I*) gene was identified as a candidate tumour suppressor gene within this chromosomal region (Kamb et al, 1994). It encodes the cyclin-dependent kinase inhibitor protein p16, a negative cell cycle regulator. Binding of p16 to CDK4 prevents the association of CDK4 with cyclin D1. Phosphorylation of substrates essential for G1 transition of the cell cycle is subsequently inhibited (Serrano et al, 1993). Inactivation of the *CDKN2A* gene may therefore lead to uncontrolled cell growth. In primary ovarian cancer, mechanisms of the *CDKN2A* gene inactivation remains unclear. Intragenic mutations and homozygous deletions were shown to be rare events (Campbell et al, 1995; Hatta et al, 1995; Shih et al, 1997). Methylation status and expression pattern were reported mainly with conflicting results (Fujita et al, 1997; Shih et al, 1997). To determine the role of the *CDKN2A* gene in the development of ovarian carcinoma, LOH at 9p21, homozygous deletion, mutation and methylation status of the *CDKN2A* gene as well as its expression at mRNA and protein levels were analysed in a panel of sporadic ovarian tumours.

### MATERIALS AND METHODS

### Samples

Samples used in this study ($n$ = 33; 23 OCs, ten benign samples) were collected from patients (informed consent) during the time of primary surgery at the Department of Obstetrics and Gynaecology, Heinrich-Heine University, Düsseldorf, Germany. Only serous epithelial carcinoma were included. Pathological grades were G1 = two, G2 = 11 and G3 = ten cases. Clinical stage of OC patients were stage I+II = nine and III+IV = 14 cases. Three benign serous cystoma and seven normal ovarian samples were also analysed. For these samples the superficial cell layers were used. None of the patients received chemotherapy or endocrine treatment prior tumour excision. Surgically removed tissues were snap frozen (liquid nitrogen, –80°C) for later extraction of DNA

*Received 16 September 1998*
*Revised 26 February 1999*
*Accepted 11 March 1999*

*Correspondence to:* D Niederacher, Molekulargenetisches Labor der Universitäts-Frauenklinik im BMFZ, Gebäude 23.12.04, Moorenstr 5, 40225 Düsseldorf, Germany

**Table 1** Primers used and PCR conditions

| Primer | Sequence | Length of PCR product (bp) | Annealing temperature (°C) | No. of cycles | Use |
|---|---|---|---|---|---|
| D9S157 | AGCAAGGCAAGCCACATTTC[a]<br>TGGGGATGCCCAAGTAACTATATC | 133–155 | 55 | 28 | LOH |
| D9S162 | GCAATGACCAGTTAAGGTTC[a]<br>AATTCCCACAACAAATCTCC | 172–196 | 55 | 28 | LOH |
| IFN-α | GTAAGGTGGAAACCCCCACT[a]<br>TGCGCGTTAAGTTAATTGGTT | 138–150 | 55 | 28 | LOH |
| D9S942 | GCAAGATTCCAAACAGTA[a]<br>CTCATCCTGCGGAAACCATT | 100–130 | 55 | 27 | LOH |
| D9S171 | AGCTAAGTGAACCTCATCTCTGTCT[a]<br>ACCCTAGCACTGATGGTATAGTCT | 159–177 | 55 | 27 | LOH |
| D9S126 | ATTGAAACTCTGCTGAATTTTCTG[a]<br>CAACTCCTCTTGGGAACTTGC | 225–250 | 56 | 34 | LOH |
| D9S169 | AGAGACAGATCCAGATCCCA[a]<br>TAACAACTCACTGATTATTTAAGGC | 259–275 | 55 | 28 | LOH |
| D9S161 | TGCTGCATAACAAATTACCAC[a]<br>CATGCCTAGACTCCTGATCC | 119–135 | 56 | 32 | LOH |
| CDKN2A<br>Exon 1 | GCGCTACCTGATTCCAATTC[a]<br>GAAGAAAGAGGAGGGGCTG | 340 | 60 | 35<br>26<br>32 | exon 1 Methyl.<br>exon 1 HD<br>exon 1 EMD |
| CDKN2A<br>Exon 2 | ACAAGCTTCCTTTCCGTCAT[a]<br>GCGCACGTCCAGCCGCGCCCCGG | 235 | 60 | 26 | exon 2 HD |
| CDKN2A<br>Exon 2 | GGAAATTGGAAACTGGAAGC<br>TCTGAGCTTTGGAAGCTCT[a] | 509 | 56 | 35 | exon 2 Methyl. |
| CDKN2A<br>Exon 2 | ACAAGCTTCCTTTCCGTCAT[a]<br>TCTGAGCTTTGGAAGCTCT[a] | 422 | 64 | 32 | exon 2 EMD |
| GAPDH 1 | TGTGCTCCCACTCCTGATTTC[a]<br>CAAAGGGCAGGAGTAAAGGTC | 200 | | | ref. for fd-PCR |
| CDKN2A<br>Expression | GCCCAACGCACCGAATAGT[a]<br>CGATGGCCCAGCTCCTCAG | 261 | 60 | 37 | expression |
| GAPDH 2 | CCATGGAGAAGGCTGGGG[a]<br>CAAAGTTGTCATGGATGACC | 195 | 60 | 27 | ref. for fd-RT-PCR |

HD, homozygous deletion; Methyl., methylation; ref., reference. [a]Fluorescent labelled.

and RNA (Niederacher et al, 1997). Haematoxylin & eosin (H&E) staining was used for routine pathological evaluation. The content of tumour cells in the samples analysed was more than 70%. As a source of normal DNA peripheral blood was used.

### LOH analysis of loci 9p21–22

Eight microsatellite markers at 9p21–22 were analysed. Primers used and polymerase chain reaction (PCR) amplification conditions are shown in Table 1. Primer sequences were obtained from the Genome Data Base (http://www.gdb.org/) and purchased from Pharmacia (Freiburg, Germany). One primer of each pair was (Cy5) fluorescent-labelled at the 5′ end. PCR products were analysed in an automated laser-activated fluorescent DNA sequencer (A.L.F. express, Pharmacia Biotech). The assessment of allele loss were performed as described previously (Niederacher et al, 1997).

### Homozygous deletion analysis of *CDKN2A* gene

Homozygous deletion (HD) of *CDKN2A* gene was determined by fluorescent differential PCR (fd-PCR) (An et al, 1995). Glyceraldehyde 3-phosphate dehydrogenase (GAPDH) was used as internal standard. PCR products were analysed in a DNA sequencer and assessment of HD was performed using AlleleLinks software (Amersham Pharmacia Biotech). Deletion status of the first and second exon of *CDKN2A* gene was analysed, respectively. Normal DNA from peripheral blood leucocytes was used as control. Reduction of the ratio *CDKN2A*:GAPDH of more than 50% compared with that in the normal controls was considered to present a HD.

### Mutation analysis of *CDKN2A* gene

Mutation status of the *CDKN2A* gene was determined by EMD (enzymatic mutation detection) (Del Tito et al, 1998) using Passport™ Mutation Scanning Kit (Amersham Pharmacia Biotech). EMD was performed according to the protocol provided by the manufacturer. Briefly, 20 µl PCR products were denatured at 95°C for 5 min followed by hybridization at room temperature for 30 min. Then, 2 µl T4 Endonuclease VII were added and cleavage was performed at 37°C for 30 min. Final products were analysed in a DNA sequencer and assessment was performed using ALFwin™ Fragment Analyser 1.01 (Amersham Pharmacia Biotech). The mutation status of the first and second exon of *CDKN2A* gene was analysed respectively. Primer used and PCR conditions are shown in Table 1.

### Methylation analysis of *CDKN2A* gene

The methylation status of CpG islands within the *CDKN2A* gene were analysed by PCR-based methylation assay

© 1999 Cancer Research Campaign 

(Gonzalez-Zulueta et al, 1995; Lo et al, 1996). Briefly, 1 µg of genomic DNA was digested by 10 U *Sma*I, *Cfo*I or *Hpa*II separately (at 30°C, 37°C, 37°C respectively) for 12 h. Thereafter, 10 U enzymes were added and digestion continued for another 12 h. Digested DNA was subjected to PCR. Methylation status of the first and second exon of *CDKN2A* gene was analysed separately. The primers used (Table 1) amplified exon 1, a 340 bp fragment including one *Sma*I, four *Cfo*I and two *Hpa*II sites, and exon 2, a 509 bp fragment including one *Sma*I, seven *Cfo*I and four *Hpa*II sites. GAPDH was co-amplified as an internal control. Digested DNA from normal tissue was used as negative control to ensure that digestion was complete. Undigested DNA was amplified as positive control.

### *CDKN2A* gene mRNA expression

Total cellular RNA was isolated using TRIZOL (Gibco-BRL, Glasgow, UK). One microgram RNA was subjected to digestion by DNAase I (Gibco-BRL, Rockville, MD, USA) according to the protocol provided by the producer. A total of 0.86 µg of the digested RNA was used for cDNA synthesis. Briefly, digested RNA was denatured at 90°C for 5 min and transcribed by Mulv-Reverse-Transcriptase (Pharmacia, Freiburg, Germany) using hexanucleotide (random-) primers. Final reaction volume of 20 µl contained 50 mM potassium chloride, 10 mM Tris–HCl, 7.5 mM magnesium chloride, 0.1 mg $ml^{-1}$ bovine serum albumin (BSA), 15 mM dithiothreitol (DTT), 1 mM each dNTP, 1 U $\mu l^{-1}$ RNAasin (Promega), 1000 pmol random-primers, 9.5 U Mulv-Reverse-Transcriptase. The conditions were: 26°C for 10 min, 42°C for 45 min and 95°C for 5 min. A 2 µl cDNA aliquot corresponding to 86 ng of RNA was used as template for the reverse transcription PCR (RT-PCR) amplification. Primers used were shown in Table 1. GAPDH was co-amplified as internal standard. To maintain both amplifications (*CDKN2A* and GAPDH) in the exponential phase a ‘drop-in’ method was used for the differential RT-PCR. Thirty-seven cycles were performed for *CDKN2A* and 27 cycles for GAPDH. PCR products were analysed in a DNA sequencer and assessment was performed as above. mRNA levels in OC were compared to that in normal ovarian tissues. Expression level below the normal range was considered as low, above the normal range as high expression.

### Immunohistochemistry

Immunohistochemical staining for *CDKN2A* gene product p16 was performed on paraffin-embedded formalin-fixed sections using the indirect avidin–biotin peroxidase method as described previously (Beckmann et al, 1996*c*). Representative paraffin blocks were sectioned at 5 µm, affixed to 0.02% poly-L-lysine-coated slides and dried. Sections were dewaxed in xylene and rehydrated through descending grade alcohol to phosphate-buffered saline (PBS) (pH 7.4), followed by digestion with 0.1% trypsin for 20 min, incubation in methanol with 0.03% hydrogen peroxide for 20 min and 30% normal horse serum 20 min at room temperature. Sections were then incubated with mouse anti-human p16 monoclonal antibody (1:300 dilution, Pharmingen, San Diego, CA, USA) overnight at 4°C. This was subsequently followed by biotinylated horse anti-mouse antibody (1:100) and by avidin–biotin–peroxidase complex (Vector Laboratories, Burlingame, CA, USA). As negative control, irrelevant mouse monoclonal IgG was applied as the primary antibody. A known p16-positive epithelial tissue sample was used as positive control. Immunoreactivity of p16 was quantified by counting staining positive and negative tumour cells in a minimal total of 1000 cells. The relative number of immunoreactive cells was graded as follows: < 10% = –, 10–50% = +, >50% = ++. Intensity of staining was not evaluated.

### Statistical analysis

Statistical analysis was performed by using SPSS (8.0) software.

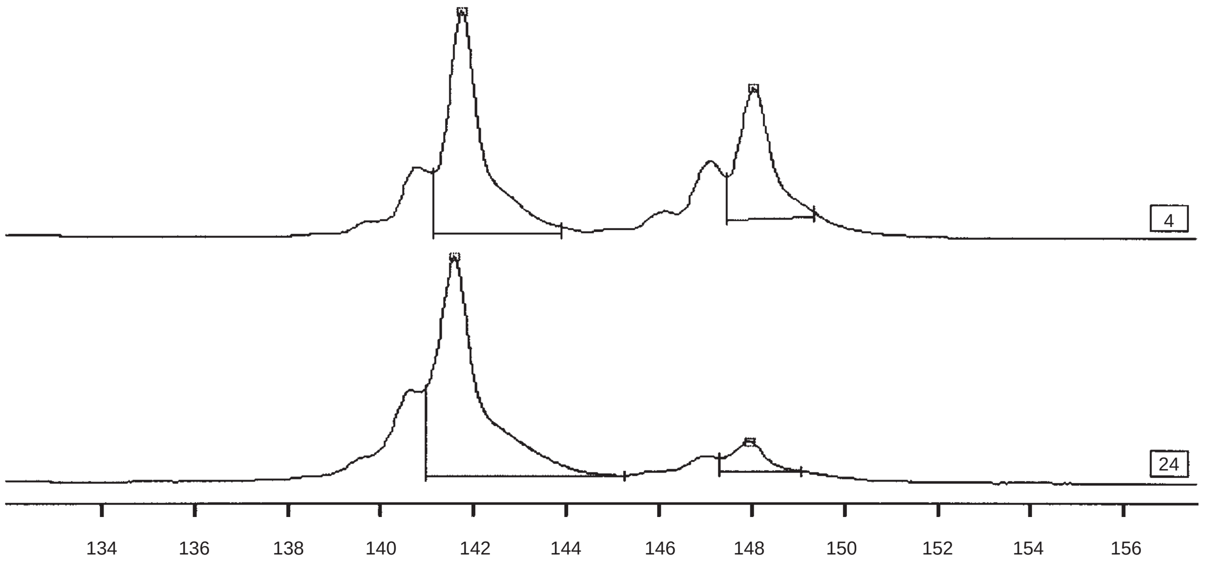

**Figure 1** LOH analysis of marker D9S162 (*x*-axis: run time (min)). Lane 4: normal blood DNA, lane 24: tumour DNA with signal reduction (80%) in the second allele

© 1999 Cancer Research Campaign

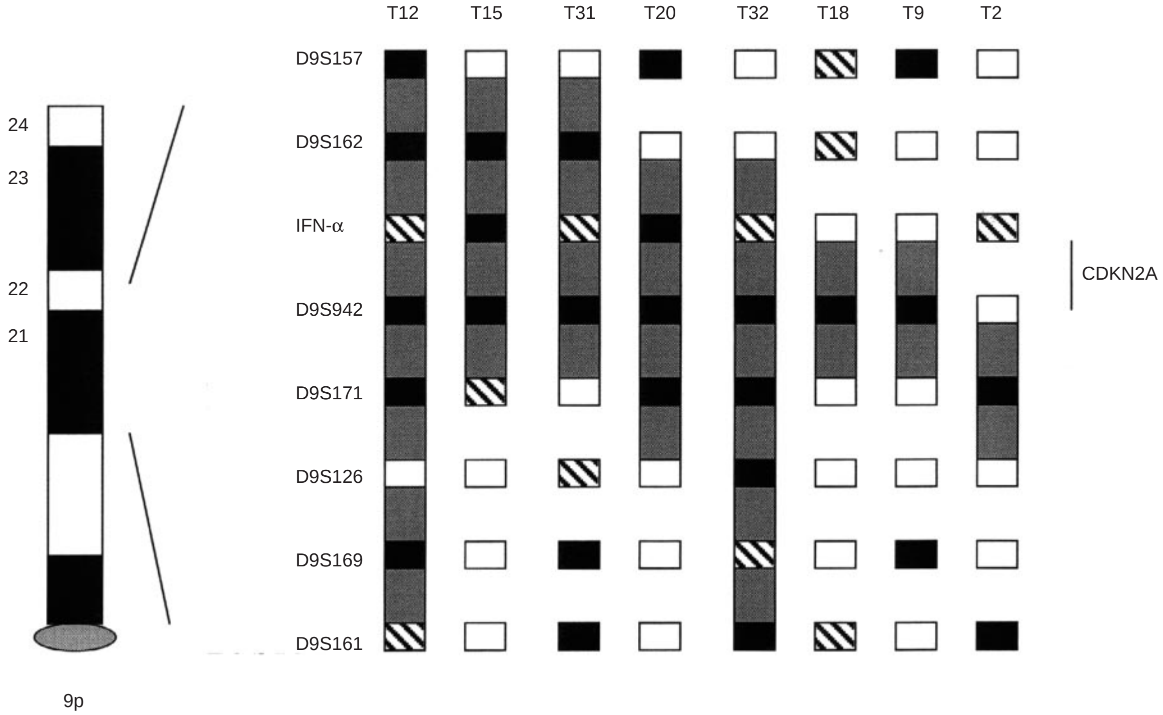

**Figure 2** Schematic representation of eight patients with partial deletions at 9p21–22. (■), LOH; (▧), not informative; (□), retention of heterozygosity; (■), largest possible region of deletion

### RESULTS

### LOH on chromosome 9p21–22

Paired normal and tumour DNA from 23 malignant tumours and three benign tumours were analysed for LOH. No LOH was found in the benign tumours. Of the 23 malignancies analysed, 15 (65%) showed LOH of at least one or more microsatellite markers. Representative examples of LOH are shown in Figure 1. Frequencies of LOH in the tumour panel varied according to the markers tested. D9S157 and D9S169 showed the least frequent (40%) and D9S942, IFN-α and D9S171 the most frequent LOH rates (59%, 53% and 53% of informative cases respectively). In seven of the 15 tumours with allele loss, all informative markers showed LOH, suggesting that in these tumours a large region of 9p21–22 was lost. Eight tumours with partial deletion pattern are illustrated in Figure 2. Most of the tumours (7/8) showed a common region of deletion between marker IFN-α and D9S171 including the *CDKN2A* gene. Only one tumour (T2) with heterozygous marker D9S942 may further restrict the SRO (smallest region of overlap) between markers D9S942 and D9S171, but marker IFN-α is not informative in this tumour (T2). The minimum LOH rate for the *CDKN2A* gene locus estimated by the extragenic markers (IFN-α and D9S942) was 39%.

### Homozygous deletion and mutation of *CDKN2A* gene

Homozygous deletion and mutation status of the *CDKN2A* gene were analysed in all the tumours. No homozygous deletion was found. There was no evidence of mutation in both exons of the *CDKN2A* gene.

### Methylation of the *CDKN2A* locus

Methylation status of exon 1 and 2 of the *CDKN2A* gene was analysed separately. Of the 33 ovarian samples analysed (23 malignant epithelial tumours, three benign tumours and seven normal ovarian tissue samples), six malignant tumours showed methylation in exon 2, but none showed methylation in exon 1. Exon 2 methylation did not correlate to pathological grade and clinical stage of the patients and had no association with reduction of mRNA and protein expression of the *CDKN2A* gene. No methylation was found in both exons in either the benign tumours or the normal ovarian tissues.

### Expression of *CDKN2A* gene at mRNA and protein level

The mRNA level of *CDKN2A* gene was examined in a total of 22 frozen ovarian cancers and seven normal ovarian tissues by differential RT-PCR (Figure 3). Of 22 tumours analysed, low expression of the *CDKN2A* gene was detected in six tumours and high expression in eight tumours. The expression of the *CDKN2A* gene was not significantly associated with histological grade and clinical stage.

The *CDKN2A* gene product p16 was investigated by immunohistochemistry in 19 ovarian cancers and seven normal ovarian tissues. In normal ovarian tissue the epithelium showed weak positive staining. Of the 19 OCs examined, 15 showed positive staining (+/++) and four tumours were negative. The immunostaining correlated with *CDKN2A* mRNA level ($P$ = 0.038, Table 2), but was not significantly associated with pathological grade and clinical stage of patients (data not shown).

*CDKN2A* gene expression (mRNA and protein) and LOH at *CDKN2A* gene locus (D9S942 and IFN-α) were not significantly associated (Table 3).

© 1999 Cancer Research Campaign

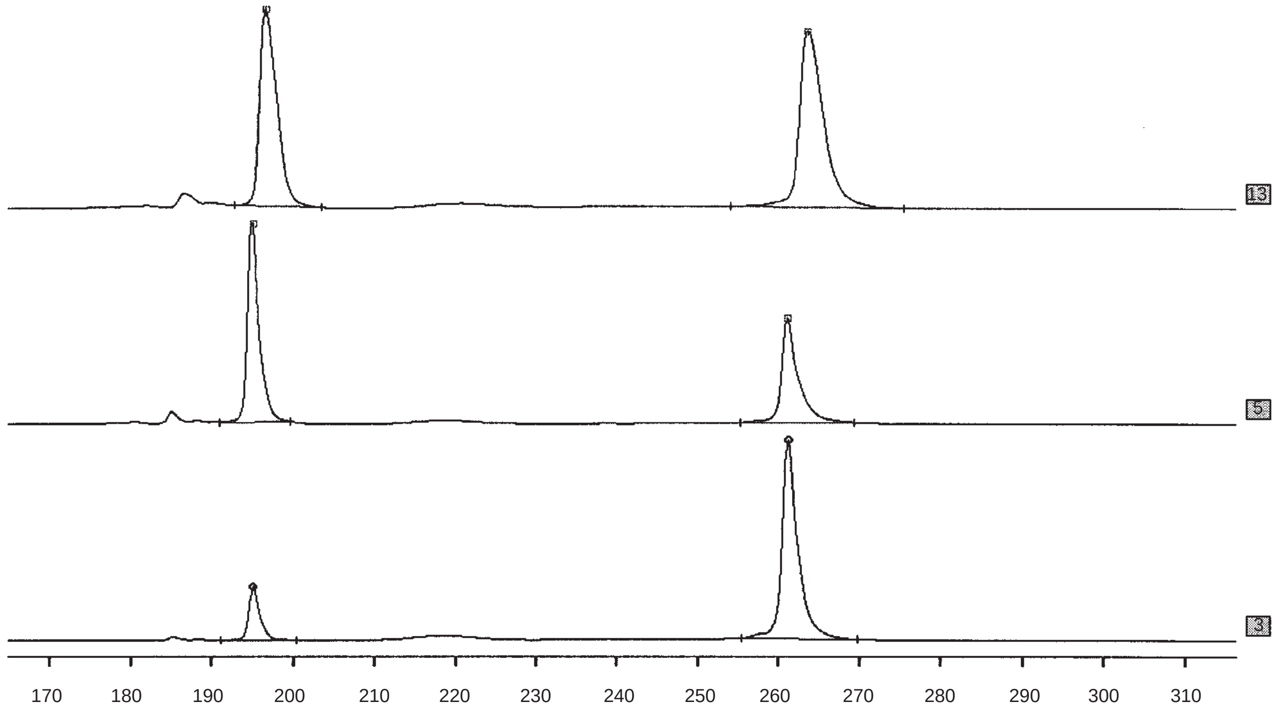

**Figure 3** *CDKN2A* expression analysis by fd-RT (*x*-axis: base pair) in ovarian cancer. First peak: reference gene GAPDH (195 bp), second peak: *CDKN2A* gene (261 bp). Lane 13: normal ovarian tissue, lane 5: ovarian cancer (T15) showed low expression (signal reduced 58% compared to normal ovarian tissues), lane 3: ovarian cancer (T10) showed high expression (signal increased three-fold compared to normal ovarian tissues)

**Table 2** Relationship between *CDKN2A* mRNA and protein expression ($n$ = 18)

| | Protein | | |
|---|---|---|---|
| | – | + | ++ |
| mRNA | | | |
| Low | 2 | 1 | 1 |
| Medium | 1 | 4 | 3 |
| High | 0 | 0 | 6 |
| | | $P$ = 0.038 | |

By $\chi^2$ test.

**Table 3** Relationship between *CDKN2A* gene expression and LOH at *CDKN2A* locus

| | mRNA | | | protein | | |
|---|---|---|---|---|---|---|
| | Low | Medium | High | – | + | ++ |
| LOH | 1 | 4 | 2 | 2 | 3 | 3 |
| No LOH | 5 | 4 | 6 | 2 | 2 | 7 |
| | | $P$ > 0.05 | | | $P$ > 0.05 | |

By $\chi^2$ test.

### DISCUSSION

LOH studies have been widely used to identify chromosome regions which may contain putative tumour suppressor genes. Frequent LOH on chromosome 9p has been observed in multiple tumour types including sporadic ovarian cancer. This indicates the presence of tumour suppressor gene(s) in this region involved in the tumorigenesis in a wide variety of tumours (Eiriksdottir et al, 1995; Kishimoto et al, 1995; Schultz et al, 1995; Devlin et al, 1996). In this study, LOH analysis at eight loci of 9p21–22 was performed in 23 serous papillary OCs by using a sensitive analytical method based on fluorescent PCR technology (Beckmann et al, 1996*b*; Niederacher et al, 1997). LOH at 9p was demonstrated in 15 of 23 (65%) tumour samples. A most common deleted region could be defined between IFN-α and D9S171; this is in good agreement with previous observations (Chenevix-Trench et al, 1994). The high frequency of LOH at 9p21 gives a strong hint to a tumour suppressor gene(s) located in this region, which is involved in the tumorigenesis of sporadic ovarian carcinomas.

The *CDKN2A* gene is the most common candidate gene in this region. It has been found to be inactivated by intragenic mutations and homozygous deletions in a variety of tumour-derived cell lines such as lung cancer, oesophagus cancer, malignant melanomas and gliomas (Chenevix-Trench et al, 1994; Kamb et al, 1994; Nobori et al, 1994). However, for most primary tumours including OC, mutations and homozygous deletions have been found to be much less frequent than in cancer cell lines (Cairns et al, 1994; Spruck et al, 1994; Campbell et al, 1995; Shih et al, 1997). More interestingly, in sporadic OC, *CDKN2A* mutations strongly associate with specific tumour subtypes. High frequency of mutation was found in mucinous and endometrioid OC, but very low in serous papillary OC (Milde-Langosch et al, 1998). Our study is in good agreement with this finding. Of 23 serous papillary OCs no mutation was found by using EMD, a novel mutation detection technique,

 *© 1999 Cancer Research Campaign*

which has been used as routine scanning method for *BRCA1/2* mutation in our laboratory. Homozygous deletion has been shown to be very rare in primary tumours including OC, regardless of the tumour subtype (Fujita et al, 1997; Milde-Langosch et al, 1998). In this study no homozygous deletion was found in 23 serous papillary OCs analysed. This further confirms that homozygous deletion does not seem to be a mechanism of *CDKN2A* gene inactivation in sporadic OC.

Beside mutation and homozygous deletion, an alternative pathway involving hypermethylation of the 5′ CpG island has been found to inactivate the *CDKN2A* gene by blocking *CDKN2A* transcription in a variety of tumours (Merlo et al, 1994; Costello et al, 1996; Lo et al, 1996). Methylation status of the *CDKN2A* gene in OC has been rarely reported and with conflicting results: no methylation (0/50) (Shih et al, 1997) and 14% (8/43) (Fujita et al, 1997). A recent study (Milde-Langosch et al, 1998) showed that methylation also seemed to be specific for tumour histological subtype. It presents much higher frequency in mucinous and endometrioid subtype than in serous papillary OC. To gain further insight to the possible role of *CDKN2A* gene methylation in ovarian carcinogenesis CpG island methylation status in exon 1 and 2 of the *CDKN2A* gene was investigated. In 23 serous papillary OCs analysed there was no evidence of methylation in exon 1. However, methylation of the second exon was found in seven cases. Methylation of exon 1 is considered to completely block the *CDKN2A* gene transcription (Merlo et al, 1995), but the mechanism of action of exon 2 methylation remains unclear (Gonzalez-Zulueta et al, 1995). In this study exon 2 methylation existed only in ovarian malignancy, but not in normal ovarian tissue and benign tumours. This implies that exon 2 methylation is to some extent involved specifically in the malignant transformation. Correlation of exon 2 methylation with gene expression level was not found suggesting that other mechanisms than transcriptional suppression may be involved.

Although conventional inactivation mechanisms of the *CDKN2A* gene, such as mutation, homozygous deletion and 5′-CpG island methylation rarely occurred in serous papillary OC, we found that absence of *CDKN2A* gene expression was not a rare event in these tumours with 27% (6/22) at mRNA level and 21% (4/19) at protein level. Among six tumours without *CDKN2A* mRNA expression, none of the known inactivation mechanisms could be detected, suggesting that other mechanisms than those examined might be involved. Interestingly, this study showed a tendency that high *CDKN2A* expression correlated to higher pathological grade and advanced clinical stage, which is in agreement with Fujita et al (1997) and Dong et al (1997). Fujita et al investigated *CDKN2A* expression in 60 OCs and concluded that loss of p16 correlated to longer survival. Dong et al showed in a much larger immunohistochemical study including 190 epithelial ovarian tumours that loss of p16 is associated with lower pathological grade, earlier clinical stage and better prognosis. Similar results were also shown by Shigemasa et al (1997) and Milde-Longosch et al (1998). These findings seem to be controversial to the negative regulatory function of p16 in cell cycle control and suggest unknown inactivating mechanisms of the *CDKN2A* gene in OC. Obviously, genomic alterations at DNA level such as deletion, mutation and methylation influence *CDKN2A* gene expression significantly. On the other hand, p16 is in a complicated feedback loop constituted by CDK4, cyclin D1, pRb and some transcriptional factors, which can regulate *CDKN2A* gene expression at transcription level (Serrano et al 1993; Kamb et al, 1994). Besides this, other bypassing pathways such as cyclin E1 is also supposed to regulate p16 expression indirectly (Gray-Babin et al, 1996). Therefore, it could be interpreted that reduced *CDKN2A* gene expression may play a key role in the early development of OC. With the progression during OC expression of the *CDKN2A* gene might be regulated by: (1) other cancer-related gene(s) involved and (2) some transcriptional factors changing with cell proliferation. Therefore, the level of *CDKN2A* gene expression is actually an indicator for the cell status. This interpretation is further supported by the result that LOH at *CDKN2A* locus does not correlate with *CDKN2A* mRNA level. *CDKN2A* gene mRNA level correlated significantly with the protein level, further indicating that post-transcriptional regulation of protein level does not seem to play a role in the *CDKN2A* expression.

Our result showed that the SRO flanked widely from D9S171 to D9S942 and IFN-$\alpha$. Besides the *CDKN2A* gene there could be other tumour suppressor gene(s) involved in the tumorigenesis of OC, such as the p15 gene or p19 gene, a novel tumour suppressor gene sharing exon 2 with the *CDKN2A* gene.

In conclusion, the results in this study suggest that there is a tumour suppressor gene(s) at 9p21 involved in the tumorigenesis of OC. The *CDKN2A* gene may be the candidate gene in this region. The inactivation of this gene showed to be tumour subtype specific. In mucinous and endometrioid OC mutation and methylation present the major inactivation mechanisms. But in serous papillary OC the inactivation mechanism remains unclear.

### ACKNOWLEDGEMENTS

The authors would like to thank the technicians of the laboratory of molecular genetics (Ms D Larbig) and of the laboratory of pathomorphology (Ms H Fröhlich; Ms U Grolik), Frauenklinik, Heinrich-Heine Universität, Düsseldorf, Germany, for the recruitment of the tumours, their expert technical assistance and persistent support.

### REFERENCES

An HX, Niederacher D, Beckmann MW, Göhring UJ, Scharl A, Picard F, van Roeyen C, Schnürch HG and Bender HG (1995) ERBB2 gene amplification detected by fluorescent differential polymerase chain reaction in paraffin-embedded breast carcinoma tissues. *Int J Cancer* **74**: 57–63

Beckmann MW, An H-X, Niederacher D, Köhrer K, Finken-Eigen M, Schröder W, Schnürch HG and Bender HG (1996*a*) Oncogene amplification in archival ovarian carcinoma detected by fluorescent differential polymerase chain reaction – a routine analytical approach. *Int J Gynecol Cancer* **6**: 291–297

Beckmann MW, Picard F, An HX, van Roeyen CR, Dominik SI, Mosny DS, Schnürch HG, Bender HG and Niederacher D (1996*b*) Clinical impact of detection of loss of heterozygosity of BRCA1 and BRCA2 markers in sporadic breast. *Br J Cancer* **73**: 1220–1226

Beckmann MW, Niederacher D, Massenkeil G, Tutschek B, Beckmann A, Schenko G, Schnürch HG and Bender HG (1996*c*) Expression analyses of epidermal growth factor receptor and HER-2/neu: no advantage of prediction of recurrence or survival in breast cancer patients. *Oncology* **53**: 441–447

Cairns P, Mao L, Merlo A, Lee DJ, Schwab D, Eby Y, Tokino K, van der Riet P, Blaugrund JE and Sidransky D (1994) Rates of p16 (MTS1) mutations in primary tumors with 9p loss. *Science* **265**: 415–416

Campbell IG, Foulkes WD, Beynon G, Davis M and Englefield P (1995) LOH and mutation analysis of CDKN2 in primary human ovarian cancers. *Int J Cancer* **63**: 222–225

Chenevix-Trench G, Kerr J, Friedlander M, Hurst T, Sanderson B, Coglan M, Ward B, Leary J and Khoo SK (1994) Homozygous deletions on the short arm of chromosome 9 in ovarian adenocarcinoma cell lines and loss of heterozygosity in tumors. *Am J Hum Gene* **55**: 143–149

© 1999 Cancer Research Campaign 

Cliby W, Ritland S, Hartmann L, Dodson M, Halling KC, Keeney G, Podratz KC and Jenkins RB (1993) Human epithelial ovarian cancer allelotype. *Cancer Res* **53**: 2393–2398

Costello JF, Berger MS, Huang HS and Cavenee WK (1996) Silencing of p16/CDKN2 expression in human gliomas by methylation and chromatin condensation. *Cancer Res* **56**: 2405–2410

Del Tito BJ Jr, Poff HE 3rd, Novotny MA, Cartledge DM, Walker RI II, Earl CD and Bailey AL (1998) Automated fluorescent analysis procedure for enzymatic mutation detection. *Clin Chem* **44**: 731–739

Devlin J, Elder PA, Gabra H, Steel CM and Knowles MA (1996) High frequency of chromosome 9 deletion in ovarian cancer: evidence for three tumour-suppressor loci. *Br J Cancer* **73**: 420–423

Dong Y, Walsh MD, McGuckin MA, Gabrielli BG, Cummings MC, Wright RG, Hurst T, Khoo SK and Parsons PG (1997) Increased expression of cyclin-dependent Chinese inhibitor 2 (CDKN2A) gene product P16INK4A in ovarian cancer is associated with progression and unfavourable prognosis. *Int J Cancer (Pred Oncol)* **74**: 57–63

Eiriksdottir G, Sigurdsson A, Jonasson JG, Agnarsson BA, Sigurdsson H, Gudmundsson J, Bergthorsson JT, Barkardottir RB, Egilsson V and Ingvarsson S (1995) Loss of heterozygosity on chromosome 9 in human breast cancer: association with clinical variables and genetic changes at other chromosome regions. *Int J Cancer* **64**: 378–382

Fujita M, Enomoto T, Haba T, Nakashima R, Sasaki M, Yoshino K, Wada H, Buzard GS, Matsuzaki N, Wakasa K and Murata Y (1997) Alteration of p16 and p15 genes in common epithelial ovarian tumors. *Int J Cancer* **74**: 148–1550

Gray-Bablin J, Zalvide J, Fox MP, Knickerbocker CJ, DeCapiro JA and Keyomarsi K (1996) Cyclin E, a redudant cyclin in breast cancer. *Proc Natl Acad Sci USA* **93**: 15215–15220

Gonzalez-Zulueta M, Bender CM, Yang AS, Nguyen T, Beart RW, Van Tornout JM and Jones PA (1995) Methylation of the 5′ CpG island of the p16/CDKN2 tumor suppressor gene in normal and transformed human tissues correlates with gene silencing. *Cancer Res* **55**: 4531–4535

Hatta Y, Hirama T, Takeuchi S, Lee E, Pham E, Miller CW, Strohmeyer T, Wilczynski SP, Melmed S, Koeffler HP Kamb A, Gruis NA, Weaver-Feldhaus J, Liu Q, Harshman K, Tavtigian SV, Stockert E and Day (1995) Alterations of the p16 (MTS1) gene in testicular, ovarian, and endometrial malignancies. *J Urol* **154**: 1954–1957

RS 3rd, Johnson BE and Skolnick MH (1994) A cell cycle regulator potentially involved in genesis of many tumor types. *Science* **264**: 436–440

Kishimoto Y, Sugio K, Hung JY, Virmani AK, McIntire DD, Minna JD and Gazdar AF (1995) Allele-specific loss in chromosome 9p loci in preneoplastic lesions accompanying non-small-cell lung cancers. *J Natl Cancer Inst* **87**: 1224–1229

Lo KW, Cheung ST, Leung SF, van Hasselt A, Tsang YS, Mak KF, Chung YF, Woo JK, Lee JC and Huang DP (1996) Hypermethylation of the p16 gene in nasopharyngeal carcinoma. *Cancer Res* **56**: 2721–2725

Merlo A, Herman JG, Mao L, Lee DJ, Gabrielson E, Burger PC, Baylin SB and Sidransky D (1995) 5′ GpG island methylation is associated with transcriptional silencing of the tumor suppressor p16/CDKN2/MTS1 in human cancers. *Natl Med* **1**: 686–692

Milde-Langosch K, Ocon E, Becker G and Loning T (1998) p16/MTS1 inactivation in ovarian carcinomas: high frequency of reduced protein expression associated with hyper-methylation or mutation in endometrioid and mucinous tumors. *Int J Cancer* **79**: 61–65

Niederacher D, Picard F, van Roeyen C, An HX, Bender HG and Beckmann MW (1997) Patterns of allelic loss on chromosome 17 in sporadic breast carcinomas detected by fluorescent-labeled microsatellite analysis. *Genes Chromosomes Cancer* **18**: 181–192

Nobori T, Miura K, Wu DJ, Lois A, Takabayashi K and Carson DA (1994) Deletions of the cyclin-dependent kinase-4 inhibitor gene in multiple human cancers. *Nature (Lond)* **368**: 753–756

Schultz DC, Vanderveer L, Buetow KH, Boente MP, Ozols RF, Hamilton TC and Godwin AK (1995) Characterization of chromosome 9 in human ovarian neoplasia identifies frequent genetic imbalance on 9q and rare alterations involving 9p, including CDKN2. *Cancer Res* **55**: 2150–2157

Serrano M, Hannon GJ, Beach D (1993) A new regulatory motif in cell-cycle control causing specific inhibition of cyclin D/CDK4. *Nature* **366**: 704–707

Shigemasa K, Hu C, West CM, Clark J, Parham GP, Parmley TH, Korourian S, Baker VV and O'Brien TJ (1997) p16 overexpression: a potential early indicator of transformation in ovarian carcinoma. *J Soc Gynecol Investig* **4**: 95–102

Shih YC, Kerr J, Liu J, Hurst T, Khoo SK, Ward B, Wainwright B and Chenvevix-Trench G (1997) Rare mutations and to hypermethylation at the CDKN2A locus in epithelial ovarian tumours. *Int J Cancer* **70**: 508–511

Spruck CH 3rd, Gonzalez-Zulueta M, Shibata A, Simoneau AR, Lin MF, Gonzales F, Tsai YC and Jones PA (1994) p16 gene uncultured tumours. *Nature (Lond)* **370**: 183–184

*© 1999 Cancer Research Campaign*
